# Calcium and IL-6 regulate the anterograde trafficking and plasma membrane residence of the iron exporter ferroportin to modulate iron efflux

**DOI:** 10.1016/j.jbc.2024.107348

**Published:** 2024-05-06

**Authors:** Shaina L. Rosenblum, Danielle K. Bailey, Daniel J. Kosman

**Affiliations:** Department of Biochemistry, Jacobs School of Medicine and Biomedical Sciences, State University of New York at Buffalo, Buffalo, New York, USA

**Keywords:** inflammation, membrane trafficking, iron metabolism, ferroportin, calcium, interleukin 6 (IL-6), metal homeostasis

## Abstract

Iron is an essential element for proper cell functioning, but unbalanced levels can cause cell death. Iron metabolism is controlled at the blood-tissue barriers provided by microvascular endothelial cells. Dysregulated iron metabolism at these barriers is a factor in both neurodegenerative and cardiovascular diseases. Mammalian iron efflux is mediated by the iron efflux transporter ferroportin (Fpn). Inflammation is a factor in many diseases and correlates with increased tissue iron accumulation. Evidence suggests treatment with interleukin 6 (IL-6) increases intracellular calcium levels and calcium is known to play an important role in protein trafficking. We have shown that calcium increases plasma membrane localization of the iron uptake proteins ZIP8 and ZIP14, but if and how calcium modulates Fpn trafficking is unknown. In this article, we examined the effects of IL-6 and calcium on Fpn localization to the plasma membrane. In HEK cells expressing a doxycycline-inducible GFP-tagged Fpn, calcium increased Fpn-GFP membrane presence by 2 h, while IL-6 increased membrane-localized Fpn-GFP by 3 h. Calcium pretreatment increased Fpn-GFP mediated ^55^Fe efflux from cells. Endoplasmic reticulum calcium stores were shown to be important for Fpn-GFP localization and iron efflux. Use of calmodulin pathway inhibitors showed that calcium signaling is important for IL-6–induced Fpn relocalization. Studies in brain microvascular endothelial cells in transwell culture demonstrated an initial increase in ^55^Fe flux with IL-6 that is reduced by 6 h coinciding with upregulation of hepcidin. Overall, this research details one pathway by which inflammatory signaling mediated by calcium can regulate iron metabolism, likely contributing to inflammatory disease mechanisms.

Iron has an essential role as a prosthetic metal. An *in silico* analysis suggests that ∼2% of human genes encode an iron-containing protein, that more than 50% of these are enzymes, and that these represent 6.5% of all enzymes expressed (1). Although essential, given its complicated redox chemistry, mismanaged iron is cytotoxic leading to cell death (2). The abluminal space in the brain and the heart are protected from circulating iron, whether transferrin-bound iron (TBI) or nontransferrin-bound iron (NTBI), by an impermeable endothelial cell barrier. Dysregulated iron metabolism within these tissues is a key factor in both neurodegenerative and cardiovascular diseases, therefore iron handling at these barriers needs to be precisely controlled (3, 4).

Iron metabolism across barriers is controlled by several proteins. The canonical TBI uptake pathway involves ferric iron (Fe^3+^)-loaded transferrin (Tf) binding to the transferrin receptor (TfR) and being taken into the cell by endocytosis. Within the low pH environment of the endosome, the Tf-loaded ferric iron is reduced to ferrous (Fe^2+^) and released from Tf/TfR, leaves the endosome, and enters the cytoplasm through the divalent metal transporter 1. Besides the canonical pathway, the divalent metal transporters ZIP8 and ZIP14 also contribute to iron uptake. These proteins can traffic iron through either TBI or NTBI. The TBI method involves ferric iron-loaded Tf binding TfR at the cell surface, reduction, and release of ferrous iron that enters the cell through ZIP8 or ZIP14. The NTBI method occurs by ferric iron being reduced and directly entering the cell through ZIP8 or ZIP14. Once iron is within the cell it can be used in several different processes or be stored in the iron storage molecule ferritin for later use (5). Iron then leaves the cell through the only known mammalian iron exporter ferroportin (Fpn) (6). Recently, evidence for another method of iron delivery across the blood brain barrier (BBB) has been found. Palsa *et al.* (7) discovered that ferritin and Tf can enter the brain through transcytosis, involving exosomes that transport these proteins bound to iron across BBB endothelial cells.

Iron metabolism and inflammation are inherently connected. When an inflammatory stimulus occurs, macrophages, and other monocytes respond by releasing cytokines. These cytokines induce the synthesis and release of acute phase proteins (APPs) from the liver. APPs function to regulate downstream responses to inflammation such as fever or leukocytosis. Interestingly, one of these downstream responses is a loss of serum iron and an increase in cellular iron load (8). There are several APPs related to iron metabolism, but the one that directly relates to this phenomenon is hepcidin. Hepcidin is a hormone induced specifically by interleukin 6 (IL-6); hepcidin binds to Fpn, inducing its internalization while targeting it for ubiquitin-dependent degradation (9, 10). As Fpn is the only protein that supports efflux of the cellular ferrous iron pool, hepcidin expression results in cell iron retention and withholding of iron from the circulation.

Inflammation is heightened in certain disease states. For example, circulating serum levels of IL-6 are increased in both type 2 diabetes and Alzheimer’s disease (11, 12). Along with circulating IL-6, brain levels of IL-6 may be heightened in certain disease states. Beta amyloid, the Alzheimer’s pathological peptide, induces IL-6 secretion from astrocytes (13). IL-6 is an important inflammatory cytokine that promotes the immune response through activating APP production, T-cell development, and B-cell antibody production. Downstream of IL-6 signaling at the cell membrane are several pathways, including Janus kinase and signal transducers and activators of transcription and PI3K/Akt pathways, leading to NFκB nuclear translocation (14). Evidence from Qiu *et al.* (15) suggests that IL-6 pretreatment enhances intracellular calcium responses to N-methyl-D-aspartate by increasing extracellular calcium influx and release of calcium from intracellular stores in cerebellar granule neurons. Another recent study showed that preproglucagon neurons in the hindbrain respond to IL-6 with increased intracellular calcium primarily coming from the extracellular space (16). These results suggest that calcium may be a downstream mediator of IL-6 signaling.

Calcium signaling also plays a role in endocytosis and protein trafficking. Research using mouse slice electrophysiology demonstrates that calcium influx triggers and increases the rate of endocytosis (17). Calmodulin bound to voltage-gated calcium channels senses calcium concentrations and initiates endocytosis. Other calcium-binding proteins, annexins, require calcium to execute their functions in protein trafficking, including early to late endosomal transport and vesicle budding from late endosomes (18, 19). Additionally, it is thought that phosphoinositides PI(3)P and PI(3,5)P_2_ bind to calcium channels in early endosomes and endolysosomes to induce calcium release, affecting vesicle membrane fusion (20, 21).

Recently our lab published evidence that calcium increases the plasma membrane localization of the manganese and iron transporters ZIP8 and ZIP14 in human brain microvascular endothelial cells, hBMVEC (22). Also, our lab has demonstrated that ZIP8/14 plasma membrane localization is reduced when inhibiting clathrin-mediated endocytosis using the dynamin inhibitor, dynasore (23). These findings suggest that calcium might alter localization of Fpn as well, thus regulating cellular iron efflux independently of hepcidin. Fpn is expressed on the surface of duodenal enterocytes, hepatocytes, macrophages, and placental cells, which are all involved with efflux of iron into the plasma (24, 25, 26). Fpn is also expressed in cells of the nervous system, including neurons and endothelial cells (27, 28), and cardiomyocytes in the heart (29). Barrier cell Fpn on the basolateral, or interstitial side, is uniquely important to iron metabolism as it is the main method of iron entry into the underlying tissue and cellular space.

In response to iron loading, Fpn traffics to the plasma membrane to support iron efflux, as shown in duodenal enterocytes and kidney proximal tube cells (30, 31). As mentioned previously, hepcidin induces endocytosis, phosphorylation, and ubiquitination of Fpn reducing its localization at the plasma membrane. After this process, Fpn traffics through the multivesicular body back to the lysosome for degradation (6, 9, 32). Flannagan *et al.* (33) demonstrated that macrophages initiate Fpn endocytosis in phagosomes to sequester pathogen iron contributing to nutritional immunity. Inhibition of SNARE proteins and the resulting localization of Fpn at the phagosome membrane determined that this process is dependent on vesicular trafficking. Also, by using a hepcidin-resistant Fpn expressed in macrophages, they determined that Fpn depletion from the membrane occurred independent of hepcidin. This suggests other methods of Fpn trafficking regulation besides the master regulator hepcidin. If Fpn membrane localization at the basolateral membrane of barrier cells is dysregulated, iron efflux will be affected resulting in either cellular iron overload or iron deficiency. Therefore, this work examines the effect of calcium and its upstream mediator, IL-6, on localization of Fpn to the plasma membrane and the subsequent cellular iron efflux.

## Results

### Calcium increases Fpn-GFP plasma membrane localization; the same response to IL-6 is delayed

**Figure 1:**
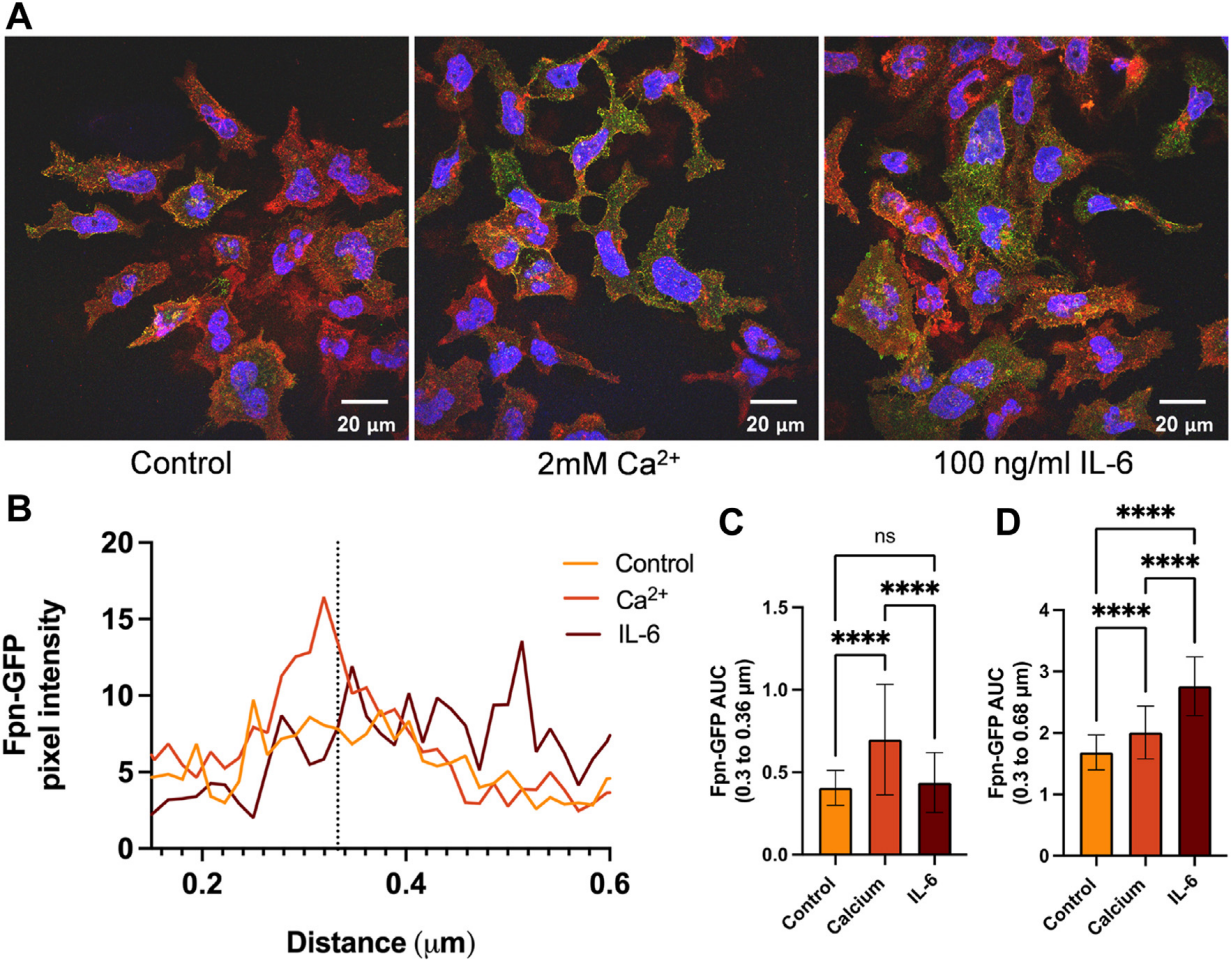
**Calcium or IL-6 treatment increases Fpn-GFP presence at the plasma membrane.** After induction of Fpn-GFP expression with 1 μg/ml doxycycline, Fpn-GFP HEK cells were treated for 1.5 h with 100 ng/ml IL-6 or 2 mM calcium. Cells on coverslips were then processed for immunocytochemistry and mounted for imaging. Fpn-GFP/Alexa Fluor 488 (*green*), plasma membrane/wheat germ agglutinin Alexa Fluor 647 (*red*), nucleus/Hoechst 33342 (*blue*). *A*, images were acquired at 63× magnification with oil immersion on a Leica TCS SP8 confocal microscope. *B*, images were quantified using ImageJ using the line analysis method described in Experimental procedures. Pixel intensities were plotted *versus* the distance along the line. The membrane peak at 0.33 μm is noted by the *vertical dotted line*. *C*, the area under the curve (AUC) was analyzed for values from 0.3 to 0.36 μm (membrane alone region) using one-way ANOVA with a Games-Howell *post hoc* test. *D*, the AUC was analyzed for values from 0.3 to 0.68 μm (postmembrane density) using one-way ANOVA with a Games-Howell *post hoc* test. The data were collected from n = 4 lines per cell (technical replicates) and n = 3 cells (biological replicates) per condition. ∗∗*p* < 0.01 and ∗∗∗∗*p* < 0.0001. Fpn, ferroportin; IL, interleukin.

To understand the effect of calcium on Fpn cellular localization, HEK293T cells expressing Fpn-GFP plated on coverslips were treated with CaCl_2_ or IL-6 for 2 h. After fixation and blocking, cells were immunostained with a GFP antibody to maximize the Fpn-GFP signal. Due to a limitation in the amount of inducible Fpn-GFP expression in these cells and potential cytotoxicity with higher doses of doxycycline, we use the GFP antibody to amplify the endogenous signal. Confocal microscopy images were taken and analyzed using a line analysis protocol outlined in the Experimental procedures section, graphing the Fpn-GFP pixel intensity across a line drawn through the membrane (Fig. 1, *A* and *B*). Area under the curve (AUC) analysis showed significant differences between the Fpn-GFP levels at the membrane in control, CaCl_2_, and IL-6–treated conditions (Fig. 1*C*). When measuring beyond the membrane, IL-6 treatment showed the largest AUC, suggesting more Fpn-GFP further inside the cell (Fig. 1*D*). Calcium pretreatment resulted in increased Fpn-GFP membrane intensity compared to the control and IL-6 conditions. Based on the line graph, at the membrane peak, the calcium condition has the highest Fpn-GFP pixel intensity, while the IL-6–treated cells have higher intensity further along the line within the cytoplasmic region, suggesting differential responses of Fpn relocalization to calcium and IL-6. These results also show that the IL-6 effect is delayed compared to calcium at 2 h, suggesting that IL-6 may be upstream of calcium signaling.

### Calcium increases plasma membrane residence of Fpn-GFP

**Figure 2:**
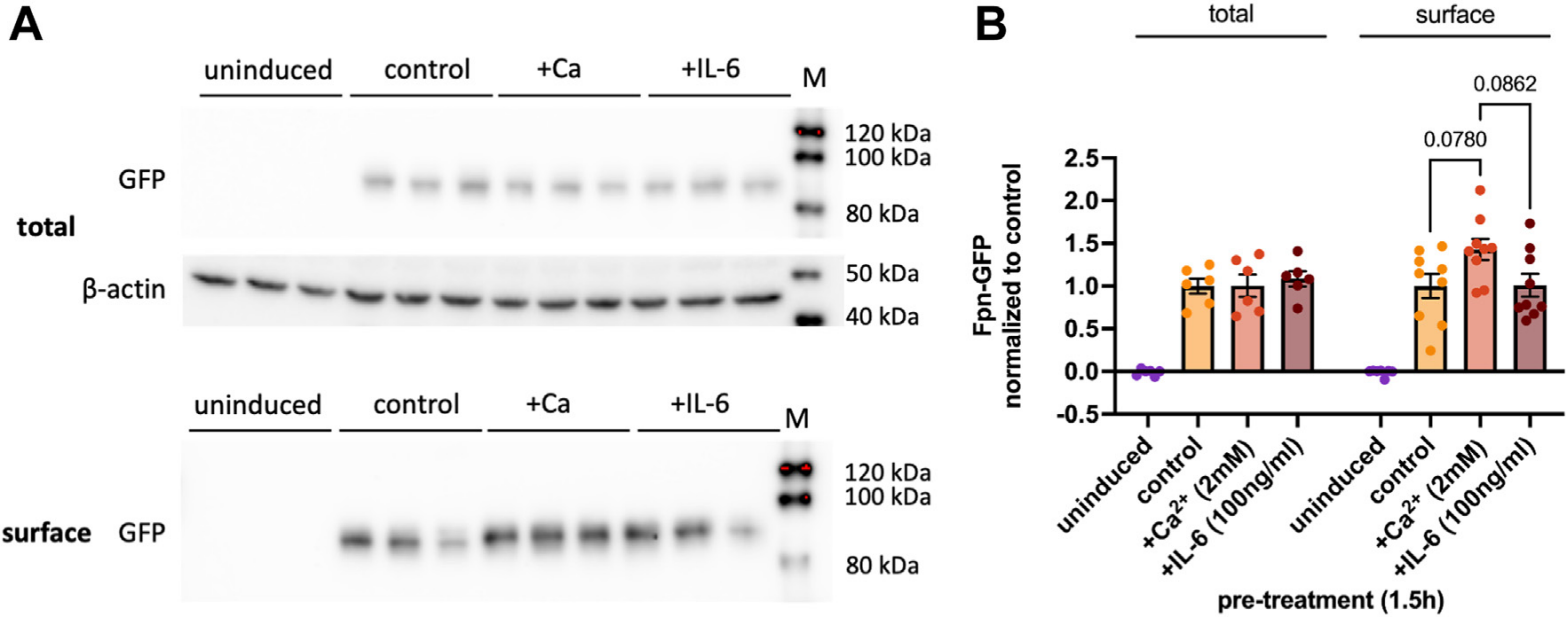
**Surface biotinylation demonstrates calcium increases plasma membrane residence of Fpn-GFP.** Fpn-GFP expression was induced in Fpn-GFP HEK cells with 1 μg/ml doxycycline. Cells were treated with 2 mM CaCl_2_ or 100 ng/ml IL-6 for 1.5 h prior to surface biotinylation. After treatment, cells were lysed, and protein content was quantified. Equal amounts of protein were loaded on Neutravidin columns and incubated overnight, then bound protein was eluted in SDS loading buffer containing 150 mM DTT. *A*, Western blots for α-GFP or α-β-actin in total and surface fractions. *B*, band intensities for GFP were normalized to those for β-actin, and quantification of the band intensities normalized to induced control are shown for both total and surface fractions. This experiment includes n = 3 trials with 3 to 6 replicates per condition. Statistical significance was tested by one-way ANOVA for total or bound fractions independently; *p* values are noted. Fpn, ferroportin; IL, interleukin; M, Magic Mark Western blot ladder.

We next evaluated total Fpn-GFP protein and its plasma membrane localization using cell surface biotinylation. Cells as above were treated with CaCl_2_ or IL-6 for 1.5 h and then incubated with succinimidyl-2-(biotinamido)-ethyl-1,3-dithiopropionate-biotin to target proteins residing on the cell surface. After lysing and protein quantification, lysates were loaded on neutravidin columns to separate the bound surface fraction from the unbound cytosolic fraction. The amount of Fpn-GFP in these fractions was then measured by Western blotting (Fig. 2*A*). No differences were found between conditions with respect to total Fpn-GFP. However, surface levels of GFP were increased by 50% with calcium compared to control although this apparent increase was not significant at the 95% confidence limit. In contrast, the 1.5 h IL-6 treatment elicited no change in Fpn-GFP surface abundance (Fig. 2*B*). This pattern paralleled the results obtained by indirect immunofluorescence with respect to the delayed Fpn-GFP relocalization following IL-6 treatment. Note the negative control, uninduced cells that expressed no detectable Fpn-GFP in the total and surface fractions (Fig. 2, *A* and *B*). Similar results were obtained with cell surface biotinylation using hBMVECs treated with CaCl_2_ (Fig. S1, *A* and *B*). These results compare with our previous finding that calcium alters localization of the uptake transporters ZIP8 and ZIP14 (22).

### Calcium treatment increases ^55^Fe efflux in Fpn-GFP HEK cells

**Figure 3:**
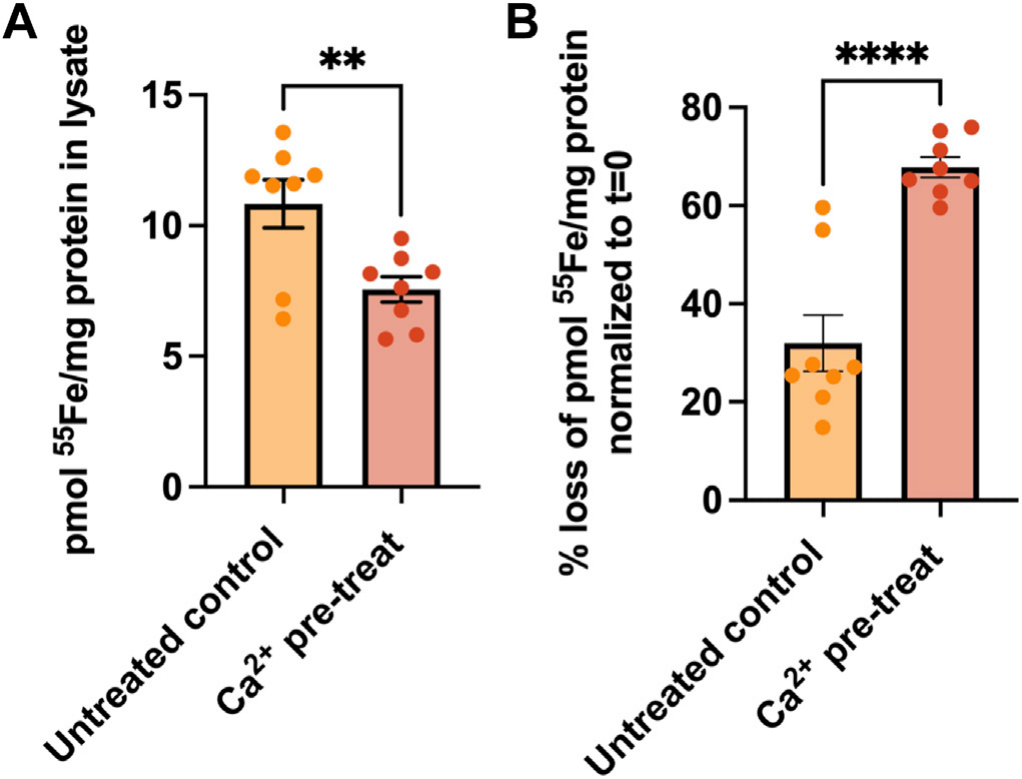
**Calcium treatment increases**^**55**^**Fe efflux in Fpn-GFP HEK cells.** Fpn-GFP expression was induced in Fpn-GFP HEK cells with 1 μg/ml doxycycline. Cells were loaded with 1 μM ^55^Fe for 24 h prior to treatment. After loading, Fpn-GFP HEK cells were treated with 1.5 h of 2 mM CaCl_2_. Media containing treatment and ^55^Fe was then removed, and efflux was conducted for 2 h. Cells were lysed and samples counted for ^55^Fe content by liquid scintillation counting. *A*, ^55^Fe content normalized to protein content is reported. *B*, percent loss of ^55^Fe from Fpn-GFP HEK is reported, normalized to protein content, and the same treatment collected at time zero of efflux. This experiment includes n = 8 biological replicates per condition. Statistical significance was tested by one-way ANOVA. ∗∗*p* < 0.01 and ∗∗∗∗*p* < 0.0001. Fpn, ferroportin.

To analyze the effect of calcium on Fpn-GFP function, ^55^Fe efflux assays were performed. Fpn-GFP induced cells were loaded with ^55^Fe for 24 h before treatment with CaCl_2_. Following treatment with CaCl_2_ for 2 h, the CaCl_2_ was removed, and cells were allowed to efflux for 2 h. After efflux, cells were lysed and counted for retained ^55^Fe by liquid scintillation counting. The data showed that Fpn-GFP cells pretreated with calcium contain significantly less pmol ^55^Fe/mg protein compared to the control condition (Fig. 3*A*). Cells pretreated with calcium exhibited a greater overall percent loss of ^55^Fe/mg protein or increased ^55^Fe efflux (Fig. 3*B*). These experiments were replicated in hBMVEC where calcium treatment amplified ^55^Fe efflux (Fig. S1, *C* and *D*).

### IL-6 stimulates a temporal increase of Fpn-GFP presence at the plasma membrane

**Figure 4:**
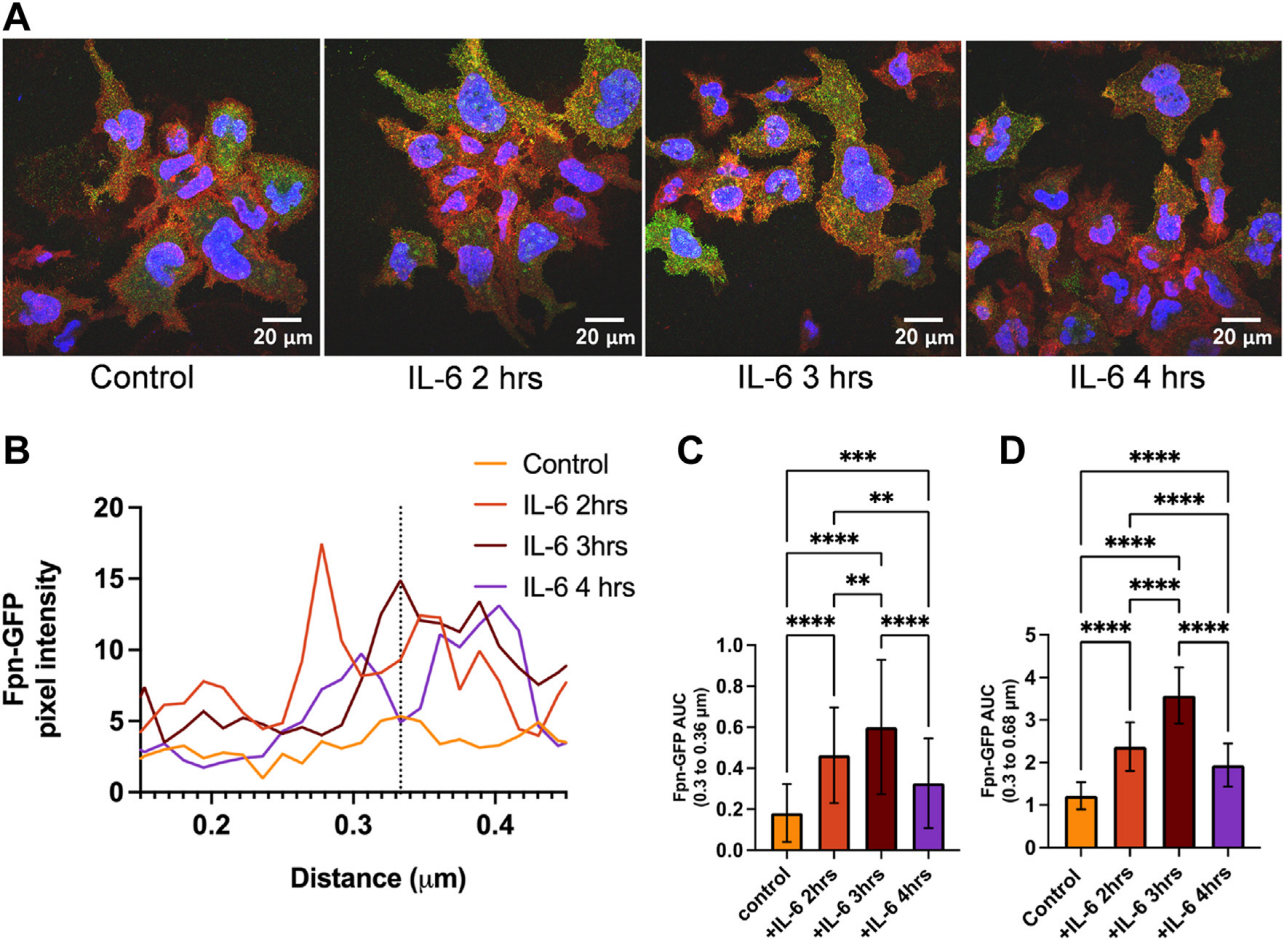
**IL-6 increases Fpn-GFP presence at the plasma membrane by 2 h that decreases in abundance by 4 h.** Fpn-GFP expression was induced in Fpn-GFP HEK cells with 1 μg/ml doxycycline. Fpn-GFP HEK cells were treated with 100 ng/ml IL-6 for 2, 3, or 4 h. Cells on coverslips were then processed for immunocytochemistry and mounted for imaging. *A*, images were acquired at 63× magnification with oil immersion on a Leica TCS SP8 confocal microscope. *B*, images were quantified using ImageJ using the line analysis method described in Experimental procedures. Pixel intensities across this line were plotted *versus* the distance of the line. The membrane peak at 0.33 μm is noted by the *vertical dotted line*. *C*, the area under the curve (AUC) was analyzed for values from 0.3 to 0.36 μm (membrane alone region) using one-way ANOVA with a Games-Howell *post hoc* test. *D*, the AUC was analyzed for values from 0.3 to 0.68 μm (membrane alone region) using one-way ANOVA with a Games-Howell *post hoc* test. The experiment was completed once with n = 4 lines per cell (technical replicates) and n = 3 cells (biological replicates) per condition. ∗*p* < 0.05 and ∗∗∗∗*p* < 0.0001. Fpn, ferroportin; IL, interleukin.

As we did not measure any differences in Fpn-GFP membrane localization when treating with IL-6 for 1.5 or 2 h, we next treated Fpn-GFP cells over a longer time period to interrogate the temporal impact IL-6 might have on Fpn localization. Fpn-GFP cells plated on coverslips were treated with IL-6 for 2, 3, or 4 h. After treatment, cells were fixed, blocked, and incubated with primary GFP antibody. Cells were then stained with secondary antibody and mounted for confocal microscopy (Fig. 4*A*). Confocal microscopy images were analyzed with the Image J (https://imagej.net/ij/) line analysis method mentioned previously. Fpn-GFP pixel intensity was plotted against distance of the line showing that at the membrane, Fpn-GFP intensity peaks by 3 h and then decreases by 4 h, consistent with retrograde protein trafficking (Fig. 4*B*). AUC analysis showed significant differences between the Fpn-GFP levels at the membrane and further inside the cell, in cells treated with IL-6 for 2, 3, and 4 h compared to control, as well as a significant difference between IL-6 2 h and 3 h and IL-6 3 h and 4 h. (Fig. 4, *C* and *D*). This behavior suggests that IL-6-dependent relocalization of Fpn to the plasma membrane is maximum at 3 h rather than 2 h as with Ca^2+^, consistent with the premise that in triggering this change in the Fpn trafficking Ca^2+^ functions downstream of IL-6.

### Inhibition of IP_3_-dependent endoplasmic reticulum Ca^2+^ release suppresses IL-6–dependent Fpn-GFP relocalization

**Figure 5:**
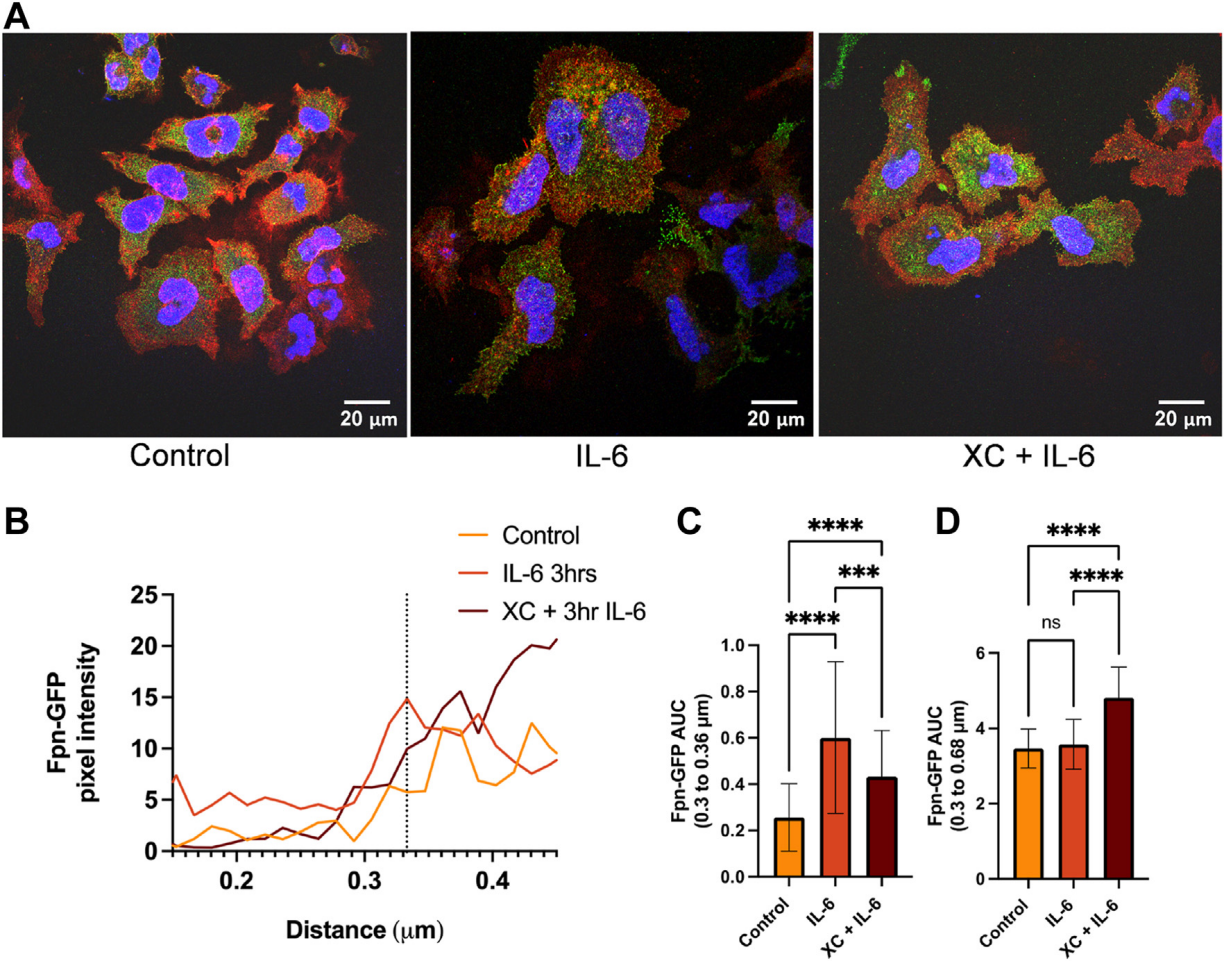
**IL-6 increases Fpn-GFP presence at the plasma membrane, which is subdued by XC treatment.** Fpn-GFP expression was induced in Fpn-GFP HEK cells was induced with 1 μg/ml doxycycline. Fpn-GFP HEK cells were treated with 3 h of 100 ng/ml IL-6 or 30 min of XC, followed by 3 h of IL-6. Cells on coverslips were then processed for immunocytochemistry and mounted for imaging. *A*, images were acquired at 63× magnification with oil immersion on a Leica TCS SP8 confocal microscope. *B*, images were quantified using ImageJ using the line analysis method described in Experimental procedures. Pixel intensities across this line were plotted *versus* the distance of the line. The membrane peak at 0.33 μm is noted by the *vertical dotted line*. *C*, the area under the curve (AUC) was analyzed for values from 0.3 to 0.36 μm (membrane alone region) using one-way ANOVA with a Games-Howell *post hoc* test. *D*, the AUC was analyzed for values from 0.3 to 0.68 μm (postmembrane density) using one-way ANOVA with a Games-Howell *post hoc* test. The experiment was completed once with n = 4 lines per cell (technical replicates) and n = 3 cells (biological replicates) per condition. ∗∗∗∗*p* < 0.0001. Fpn, ferroportin; IL, interleukin; XC, xestospongin C.

To test the premise that the IL-6 and Ca^2+^ effects were linked, and to evaluate where the calcium originates, cells were treated with xestospongin C (XC). XC is an inositol 1,4,5-trisphosphate (IP3) receptor antagonist. The IP3 receptor transports calcium out of the endoplasmic reticulum (ER). When the IP3 receptor is inhibited, cytoplasmic calcium levels are reduced (34, 35). Therefore, blocking this ER Ca^2+^ release alongside IL-6 treatment would determine if the IL-6 effect on Fpn localization is dependent on ER calcium release. Cells plated on coverslips were treated with either IL-6 alone for 3 h or following a 30 min pretreatment with XC. Confocal microscopy images were taken and analyzed using the line analysis protocol from Image J as described above. With IL-6 alone, membrane Fpn-GFP pixel intensity is the highest at 3 h. In contrast, following pretreatment with XC, PM localization is decreased associated with an apparent increase further inside the cell (Fig. 5, *A* and *B*). AUC analysis determined significant differences between the Fpn-GFP intensity at the membrane between control, IL-6 alone, and the XC + IL-6 conditions, as well as when measuring from 0.3 to 0.68, further inside the cell. IL-6 alone treatment increased Fpn-GFP AUC, which was significantly decreased by pretreatment with XC (Fig. 5*C*). Combining the XC pretreatment with IL-6 increased the AUC when examining the range from 0.3 to 0.68, suggesting greater Fpn-GFP further inside the cell upon inhibition of ER calcium release (Fig. 5*D*). This experiment links IL-6 with calcium coming from the ER and is consistent with the conclusion that the relocalization of Fpn induced by IL-6 is calcium-dependent.

### IL-6 increases ^55^Fe efflux in Fpn-GFP HEK cells, which is blocked by XC treatment

**Figure 6:**
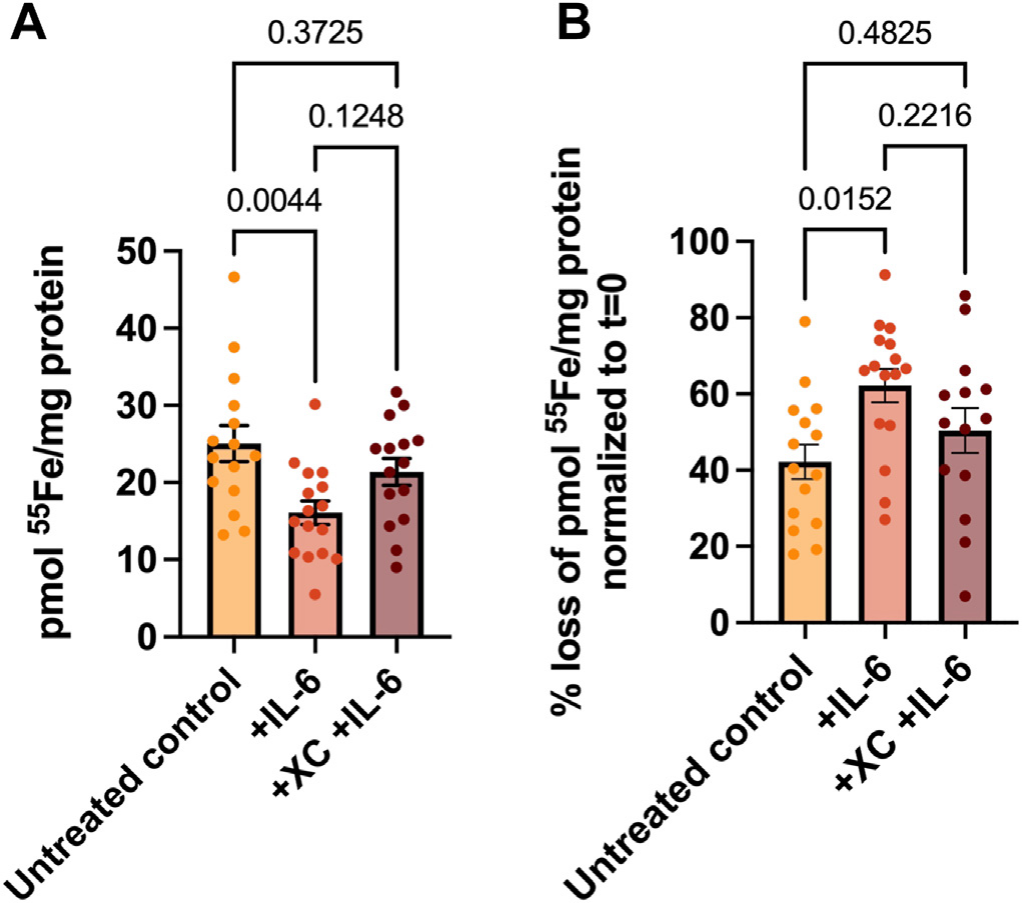
**IL-6 increases**^**55**^**Fe efflux in Fpn-GFP HEK cells, which is rescued by XC treatment.** Fpn-GFP expression was induced in Fpn-GFP HEK cells with 1 μg/ml doxycycline. Cells were loaded with 1 μM ^55^Fe for 24 h prior to efflux. Cells were pretreated with 100 ng/ml IL-6 for 3 h or for 30 min with XC, followed by 3 h of IL-6 prior to initiation of efflux. Media containing treatments and ^55^Fe was then removed, and cells were incubated for efflux for 2 h. After 2 h, cells were lysed and samples counted for ^55^Fe content by liquid scintillation counting. *A*, ^55^Fe content remaining in cell lysates, normalized to protein content. *B*, percent loss of ^55^Fe from cells is reported, normalized to protein content, and represented as percent of t = 0 for that treatment group. This experiment includes n = 2 trials with eight replicates per condition. Statistical significance was tested by one-way ANOVA. Associated *p*-values are given in the figure. Fpn, ferroportin; IL, interleukin; XC, xestospongin C.

Subsequently, iron efflux from Fpn-GFP expressing cells was functionally examined in the presence of IL-6 or XC + IL-6. As above, cells were loaded with ^55^Fe for 24 h prior to efflux. Cells were pretreated with IL-6 alone for 3 h or XC for 30 min, followed by IL-6 for 3 h. After these treatments, media was removed, and cells were allowed to efflux for 2 h. Cells were lysed and counted for ^55^Fe content by liquid scintillation counting. With IL-6 alone, significantly less ^55^Fe remained in the cell lysate compared to control, indicative of increased efflux. When treating with XC and IL-6, ^55^Fe retention in the lysate was slightly greater (Fig. 6*A*). Alternatively, when treating with IL-6 alone, there was a significantly greater percent loss of ^55^Fe, which was reduced by treatment with XC (Fig. 6*B*). This experiment functionally mirrored the results seen with confocal microscopy, consistent with the conclusion that ER calcium is involved in the plasma membrane localization of Fpn triggered by IL-6.

### Calcium signaling inhibitors reduce the IL-6–dependent increase in Fpn-GFP at the plasma membrane

**Figure 7:**
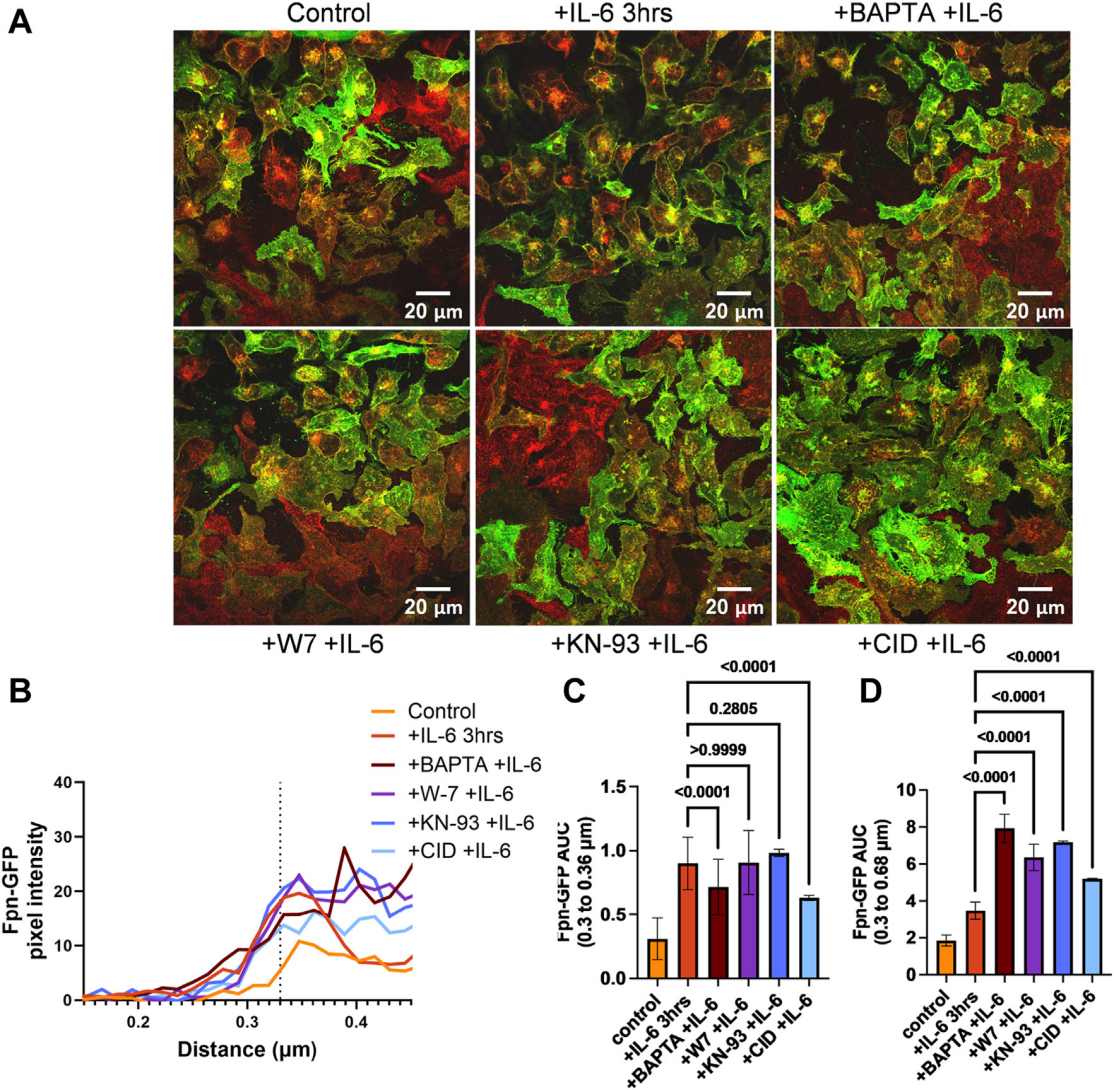
**IL-6 increases Fpn-GFP presence at the plasma membrane, which is subdued by calcium signaling inhibitors.** Fpn-GFP expression was induced in Fpn-GFP HEK cells with 1 μg/ml doxycycline. Fpn-GFP HEK cells were treated with 3 h of 100 ng/ml IL-6 or 30 min of either BAPTA, W-7, KN-93, or CID, followed by 3 h of IL-6. Cells on coverslips were then processed for immunocytochemistry and mounted for imaging. *A*, images were acquired at 63× magnification with oil immersion on a Leica TCS SP8 confocal microscope. *B*, images were quantified using ImageJ using the line analysis method described in Experimental procedures. Pixel intensities across this line were plotted *versus* the distance of the line. The membrane peak at 0.33 μm is noted by the *vertical dotted line*. *C*, the area under the curve (AUC) was analyzed for values from 0.3 to 0.36 μm (membrane alone region) using one-way ANOVA with a Games-Howell *post hoc* test. *D*, the AUC was analyzed for values from 0.3 to 0.68 μm (postmembrane density) using one-way ANOVA with a Games-Howell *post hoc* test. The experiment was completed once with n = 4 lines per cell (technical replicates) and n = 3 cells (biological replicates) per condition. ns, not statistically significant; ∗*p* < 0.05; ∗∗*p* < 0.01; and ∗∗∗∗*p* < 0.0001. BAPTA, 1,2-bis(o-aminophenoxy)ethane-N,N,N′,N′-tetraacetic acid); Fpn, ferroportin; hBMVEC, human brain microvascular endothelial cell; IL, interleukin.

To further test the premise that IL-6 and Ca^2+^ signaling were linked, cells were treated with different blockers of calcium-dependent protein trafficking. First, 1,2-bis(o-aminophenoxy)ethane-N,N,N′,N′-tetraacetic acid) (BAPTA), a well-known calcium chelator, was used to interrogate how the chelation of cytoplasmic calcium, while still treating with IL-6, altered Fpn membrane localization. Second, W-7, a calmodulin antagonist (36), and KN-93, an inhibitor of CaMKII (37), were used to determine whether the calmodulin pathway could be involved in Fpn trafficking. Last, CID-1067700, a *pan*-GTPase inhibitor (38), was used to inhibit a potential Rab GTPase downstream of calmodulin signaling. This latter choice reflected our premise that IL-6 stimulates forward-flow trafficking of Fpn naturally dependent on a Rab protein. Cells plated on coverslips were treated with either IL-6 alone for 3 h or following a 30-min pretreatment with BAPTA, W7, KN-93, or CID. Confocal microscopy images were taken and analyzed using the line analysis protocol from Image J as described above (Fig. 7, *A* and *B*). With IL-6 alone, membrane Fpn-GFP pixel intensity peaks around the denoted membrane line and decreases soon after. In contrast, following pretreatment with BAPTA, W7, KN-93, or CID, a Fpn-pixel intensity increase adjacent to the membrane was observed but had a more cytoplasmic localization. This difference is consistent with the premise that in the presence of any one of these reagents, transporter trafficking was delayed. In all cases, an increase in total Fpn-GFP was quantified by AUC analysis of the membrane proximal region and the membrane to cytoplasmic region (Fig. 7, *C* and *D*). However, in the presence of the inhibitors, this Fpn-GFP was localized more cytoplasmically than the IL-6 alone control which exhibited a primarily membrane localization. The peak amplitude of the inhibitor-treated conditions was elevated in the cytoplasmic region (∼0.4 μm), compared to the IL-6 treatment alone, suggesting that the rate of Fpn trafficking toward the membrane is slower when calcium signaling pathways were inhibited. The *p* values for each comparison are shown in a table within the supplemental material (Tables S1 and S2). These results are consistent with the premise that the relocalization of Fpn induced by IL-6 is calcium-dependent and implicate important calcium-binding and vesicular-trafficking and signaling components in this process.

### IL-6 alters temporal ^55^Fe flux across a hBMVEC barrier

**Figure 8:**
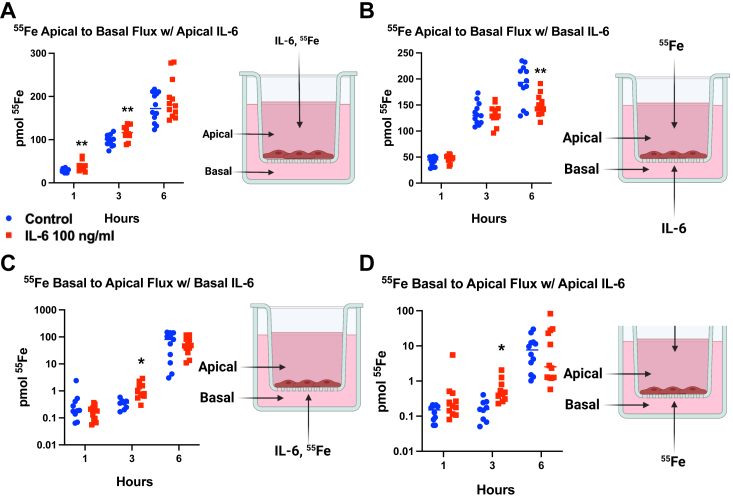
**IL-6 increases**^**55**^**Fe flux by 3 h, that returns to control by 6 h.** hBMVEC were cultured in transwells and treated with control media or IL-6 in the apical chamber (*A* and *D*) or the basal chamber (*B* and *C*) for 2 h. Radioactive ^55^Fe was added to the apical (*A* and *B*) or basal chamber (*C* and *D*) of the transwell and media was collected from the basal or apical chamber after 1, 3, and 6 h. Chamber media was counted for ^55^Fe using a liquid scintillation counter. Each panel experiment was completed once with n = 12 biological replicates per condition. Statistical analysis was done using *t* test for each time point compared to control. ∗*p* < 0.05 and ∗∗*p* < 0.01. Created with Biorender.com. Fpn, ferroportin; hBMVEC, human brain microvascular endothelial cell; IL, interleukin.

To functionally model iron flux at an endothelial cell barrier, we used hBMVEC, an immortalized cell that when grown in transwell culture dishes models the BBB. These transwell inserts separate the well into an apical (blood) chamber and a basolateral (brain) chamber. In separate flux experiments hBMVEC were treated in either the apical or basal chamber with 2 h of IL-6, to model inflammatory signals coming from the circulation or the brain interstitium, respectively. Subsequently, ^55^Fe was added either to the apical chamber or basal chamber and cells were allowed to flux iron across this barrier for 6 h. Media from either the basal chamber (apical to basal flux) or apical chamber (basal to apical flux) was collected and measured for ^55^Fe content by liquid scintillation counting. Iron flux is presented graphically as pmol ^55^Fe accumulated in the opposing chamber over this time period. At 1 h and 3 h with apical IL-6 treatment and apical to basal flux measurement (Fig. 8*A*), ^55^Fe increased in the basal chamber when treating with IL-6 compared to control. In basal to apical flux measurements, both basal IL-6 treatment (Fig. 8*C*) and apical IL-6 treatment (Fig. 8*D*) increased ^55^Fe in the apical chamber compared to control at 3 h only. In all three of these experiments (Fig. 8, *A*, *C*, and *D*) there are no differences between control and IL-6 treated with respect to ^55^Fe flux across the barrier at 6 h. In contrast, in the experiment with basal IL-6 treatment and basal to apical flux (Fig. 8*B*), there is no difference in flux with IL-6 treatment at 1 and 3 h, but a significant decrease in flux by 6 h. These experiments indicate an initial response to inflammatory signals including enhanced endothelial cell accumulation followed by efflux of iron, returning to control levels at 6 h.

### IL-6 induces hepcidin transcript abundance in hBMVEC

**Figure 9:**
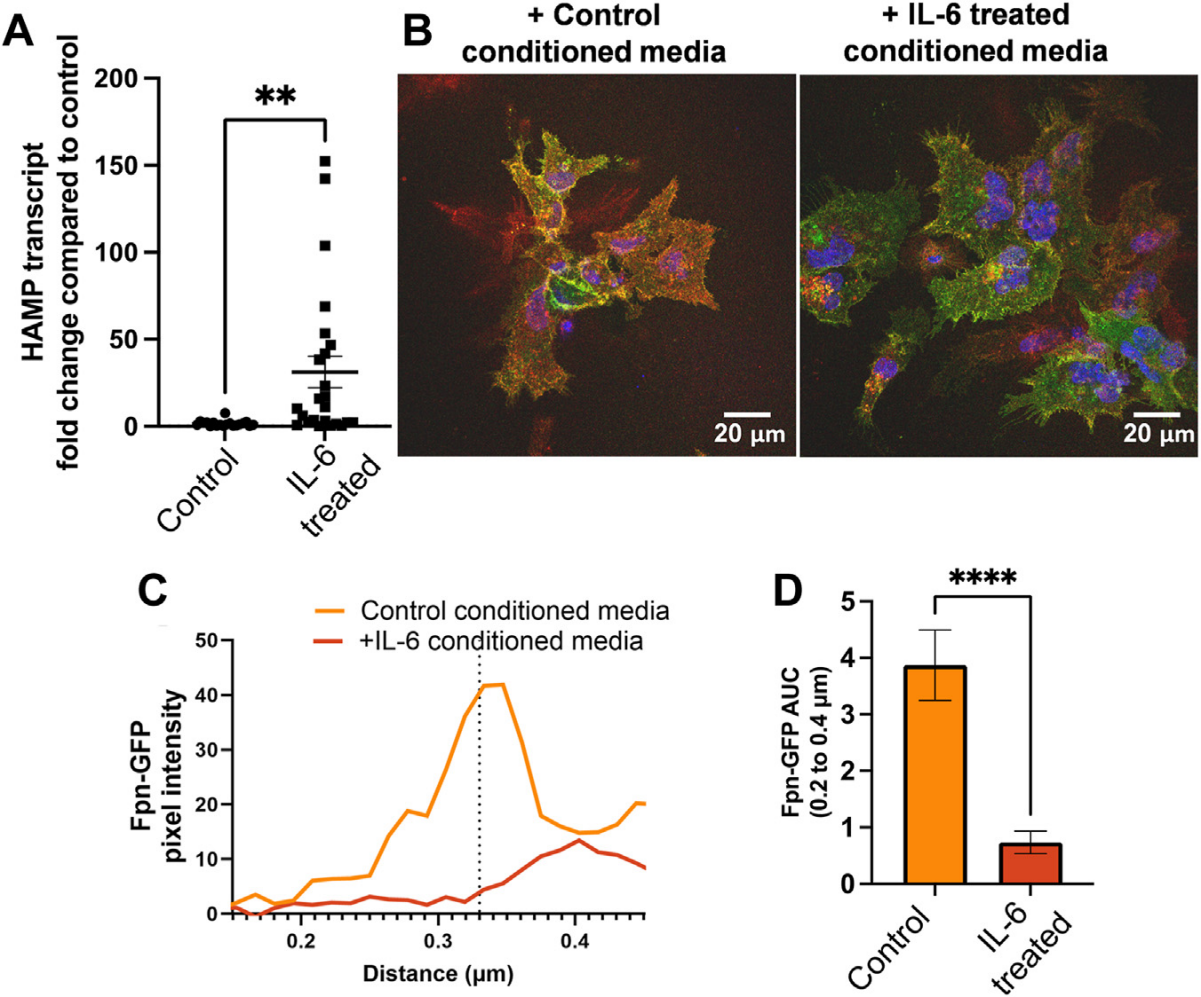
**IL-6 conditioned hBMVEC media reduces Fpn-GFP membrane presence in Fpn-GFP HEK cells.***A*, hBMVEC were grown to confluency and treated with 100 ng/µl IL-6 for 2 h, RNA was collected and reverse-transcribed, and the resulting cDNA was quantified by qPCR. Hepcidin transcript abundance in each sample was normalized to β-actin transcript, and the results are represented as the mean ± SEM fold change relative to control cells. hBMVEC were cultured to confluency in monolayers and treated with control RPMI or RPMI containing 100 ng/ml IL-6 for 2 h. After 2 h, treatments were removed and replaced with RPMI without IL-6. This media was collected after 6 h. Fpn-GFP HEK cells were plated on coverslips and Fpn-GFP expression was induced by 1 μg/ml doxycycline treatment. Conditioned media collected from hBMVEC was added to Fpn-GFP HEK cells for 3 h. Cells on coverslips were then processed for immunocytochemistry and mounted for imaging. *B*, images were acquired at 63× magnification with oil immersion on a Leica TCS SP8 confocal microscope. *C*, images were quantified using ImageJ using the line analysis method described in Experimental procedures. Pixel intensities across this line were plotted *versus* the distance of the line. The membrane peak at 0.33 μm is noted by the *vertical dotted line*. *D*, the area under the curve (AUC) was analyzed for values from 0.2 to 0.4 μm and quantified using *t* test. The experiment was completed once with n = 4 lines per cell (technical replicates) and n = 3 cells (biological replicates) per condition. ∗∗*p* < 0.01 and ∗∗∗∗*p* < 0.0001. Fpn, ferroportin; hBMVEC, human brain microvascular endothelial cell; IL, interleukin; RPMI, Roswell Park Memorial Institute.

We considered that hBMVEC hepcidin induction by IL-6 led to the decrease in ^55^Fe efflux noted directly above. To investigate this premise, hBMVEC were treated for 2 h with IL-6, RNA was collected, and qPCR was performed to assess hepcidin (HAMP) transcript levels. Indeed, IL-6 induced HAMP expression in hBMVEC compared to control cells (Fig. 9*A*).

### IL-6 conditioned hBMVEC media reduces Fpn-GFP membrane presence in Fpn-GFP HEK cells

To further interrogate the possible hepcidin effect in response to IL-6 in the flux experiment, hBMVEC were plated in monolayers and treated with 100 ng/ml IL-6 for 2 h to induce hepcidin. After the 2 h, IL-6 was removed and replaced with fresh media, incubated for an additional 6 h and the conditioned media was collected. Fpn-GFP HEK cells were plated on coverslips and Fpn-GFP expression was induced. Cells were then incubated with either untreated control or IL-6–treated hBMVEC conditioned media for 3 h. Cells were prepared for confocal microscopy, imaged, and line analysis was completed as described previously. In the control conditioned media images (Fig. 9*A*), Fpn-GFP presence on the membrane is higher than control images in other imaging experiments throughout this paper. This is likely due to the effects of treatment with conditioned media from the endothelial cells, for example, factors in the endothelial cell secretome may have contributed to increased Fpn-GFP membrane presence. However, with respect to the objective of this experiment, the Fpn-GFP cells treated with IL-6–treated hBMVEC conditioned media had lower Fpn-GFP pixel intensity at the plasma membrane than those treated with the control hBMVEC media (Fig. 9, *B* and *C*). Also, compared to control conditioned media, IL-6 conditioned media resulted in a lower AUC, suggesting that there was less Fpn-GFP in these cells overall (Fig. 9*D*). Given that IL-6 induced HAMP expression and that this conditioned media decreases Fpn presence at the membrane, these results are consistent with the model that IL-6–induced hepcidin plays a role in the loss of membrane-localized Fpn and decreased iron flux across the hBMVEC barrier.

### Basal IL-6 decreases ^55^Fe flux, which is increased by hepcidin antagonist, fursultiamine

**Figure 10:**
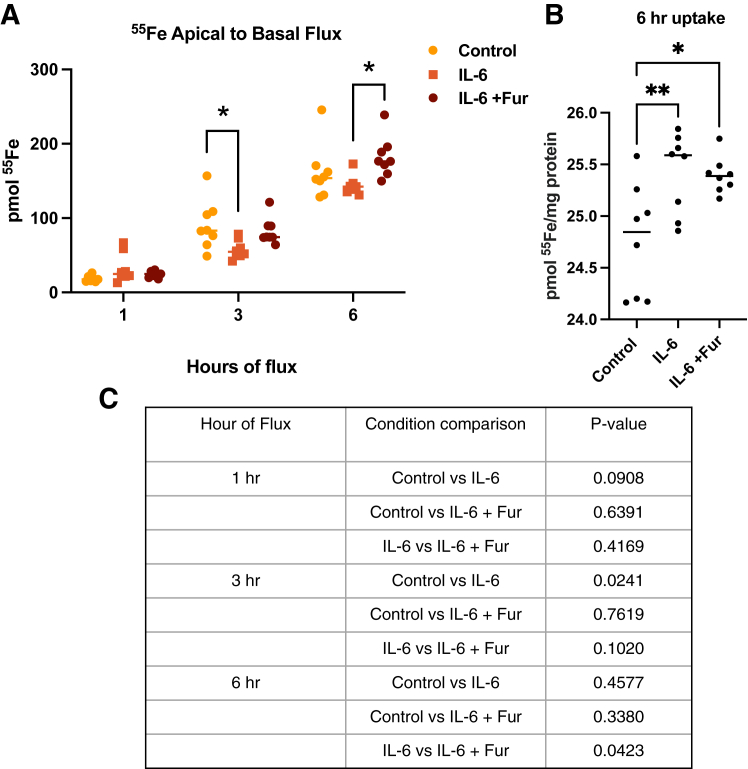
**Basal IL-6 decreases**^**55**^**Fe flux, which is recovered by hepcidin blocker fursultiamine.** hBMVEC were cultured in transwells and treated with control media or IL-6 in the basal chamber for 2 h. After 2 h, IL-6 media was removed and either replaced with control media or media containing 10 μM fursultiamine. ^55^Fe was added to the apical chamber of the transwell and media was collected from the basal chamber after 1, 3, and 6 h. Basal chamber media was counted for ^55^Fe using a liquid scintillation counter. *A*, after 6 h flux, cell lysates were collected and measured for ^55^Fe uptake as well. These values were normalized to protein content. *B*, the *p* values for the flux assay are displayed in the table in panel *C*. The experiment was completed once with n = 8 biological replicates per condition. Statistical analysis was done using *t* test within each time point compared to control. ∗*p* < 0.05 and ∗∗*p* < 0.01. Fpn, ferroportin; hBMVEC, human brain microvascular endothelial cell; IL, interleukin.

We performed an additional flux experiment to interrogate the involvement of hepcidin in this model. hBMVEC were plated in transwells and allowed to form a barrier. Cells were either untreated, treated with IL-6 in the basal chamber for 2 h, or treated with basal IL-6 for 2 h, followed by subsequent treatment with fursultiamine during the flux experiment. After the IL-6 pretreatments, ^55^Fe was added to the apical chamber and media from the basal chamber was collected at 1 h, 3 h, and 6 h to measure ^55^Fe flux. By 3 h, flux was significantly decreased in the IL-6 alone condition compared to untreated cells. In contrast, a noticeable albeit nonstatistically significant increase in flux was measured with fursultiamine compared to IL-6 treatment alone. By 6 h this fursultiamine effect was more pronounced and statistically significant (Fig. 10, *A* and *C*). In addition to the quantification of the iron flux, ^55^Fe content was measured in lysates after 6 h. There was increased ^55^Fe/mg protein in the IL-6–treated cells compared to control cells and a slight decrease when treating with fursultiamine compared to IL-6 alone, although not statistically significant (Fig. 10*B*). In total, these results suggest that basal or brain-derived IL-6 can affect endothelial cell hepcidin levels altering the flux of iron across the brain endothelial barrier.

## Discussion

With this work, we provide evidence that cytoplasmic calcium downstream of IL-6 regulates Fpn plasma membrane residence. Treatment with calcium and IL-6 increases the amount of Fpn at the HEK cell plasma membrane. We found that the IL-6 effect lags behind calcium itself with respect to Fpn relocalization. A plausible interpretation of this is that IL-6 is upstream of the calcium modulation of Fpn relocalization. This result is corroborated by surface protein biotinylation assays demonstrating increased Fpn surface presentation following calcium treatment, which does not occur within the same time frame following IL-6 application. A 1.5 h treatment was chosen based on assays and results previously published by our group (22), but it is possible that a 2 h treatment would result in greater Fpn membrane residence with calcium. One note of importance is that the total cellular amount of Fpn does not change, similar to what our lab found with respect to the uptake transporter ZIP14 (22). The data indicate that cellular localization of these two transporters, but not their abundance, is being affected.

Our imaging analysis showed that relocalization of Fpn is greatest at 3 h following IL-6 treatment. Our lab has previously shown by surface biotinylation that 3 h treatment with LPS significantly increased ZIP14 plasma membrane residence (23). As LPS is known to induce IL-6 release (39), it is likely that these two inflammatory signals regulate localization in a similar time frame. Cytokines and an inflammatory state have been shown to aid in the trafficking of proteins to the cell membrane. In neuronal SH-SY5Y cells, IL-6 treatment translocated vesicles containing the GLUT4 glucose transporter to the plasma membrane and increased glucose uptake (40). In the face of cytokine exposure, pancreatic beta cells relocate an ER chaperone GRP78 to the plasma membrane where it acts as a proapoptotic initiator (41). Interestingly, Stellwagen *et al.* (42) found that TNFα amplified exocytosis of excitatory AMPA receptors while also causing endocytosis of inhibitory gamma-aminobutyric acid receptors, providing further evidence that cytokines alter protein trafficking processes to benefit cellular homeostasis.

In support of the premise that calcium increases Fpn relocalization to the membrane, pretreatment with calcium-activated iron efflux, significantly reducing the amount of iron in cell lysates. This functionally links calcium to Fpn and iron efflux, as Fpn is the only known mammalian iron exporter (43). Work by Deshpande *et al.* (44) found that calcium is a cofactor for iron efflux, binding to Fpn and inducing a confirmational change in the protein. Another group recently corroborated a calcium-binding site in Fpn and also showed evidence that calcium can be transported by Fpn (45). Our work is independent, yet complementary, to these findings as these previous studies focused on effects of extracellular calcium on the transporter directly, while we focus on the modulation of the cellular processes affecting intracellular calcium and transporter trafficking.

Our results place importance on calcium coming from intracellular stores, specifically the ER. When blocking the ER-associated IP3 receptor, effectively decreasing cytoplasmic calcium, IL-6 had less of an effect on Fpn membrane localization. There are several examples of ER calcium–altering protein trafficking. Transient receptor potential channels are calcium permeable ion channels that are trafficked to the membrane in response to calcium coming from the ER through the Orai1 channel, known as store operated calcium entry (46, 47). In renal collecting ducts, aquaporin-2 water permeable channels are triggered to traffic to the membrane in response to vasopressin. Mechanistically, vasopressin was shown to mobilize calcium from ryanodine receptors on the sarcoplasmic reticulum of inner medullary cells (48). Work from our lab examined the role of the calcium ATPase secretory pathway Ca(2+) -ATPase pump type 1, which imports calcium into the Golgi, on trafficking of iron uptake transporters. In secretory pathway Ca(2+) -ATPase pump type 1 knockdown cells, there was a measured increase in cytosolic calcium. We further connected treatment with calcium to greater membrane localization of the iron uptake transporters ZIP8 and ZIP14 (22). Overall, our data highlights the impact of intracellular calcium stores on iron transporter plasma membrane localization.

To further understand the connections between IL-6 and calcium signaling, we interrogated effects of inhibitors of calcium-related pathways. The calmodulin pathway was chosen as it has been shown to be involved in protein trafficking processes. Calmodulin is the most ubiquitous calcium-binding protein and evidence for its involvement in the movement of proteins is abundant (49, 50, 51). Interestingly, components of the calmodulin pathway (CAMKK2 and downstream CAMK4) can regulate Tf-TfR trafficking and iron homeostasis. Sabbir demonstrated that loss of CAMKK2 and CAMK4 in mice resulted in loss of Tf trafficking and subsequently less iron content in several brain regions (52, 53). Without these important calmodulin pathway components, Tf uptake and internalization as well as Tf–TfR complex formation was decreased. Based on our evidence, Fpn trafficking and membrane localization is also dependent on these calcium-related pathways. Lastly, to further interrogate IL-6, calcium, and protein trafficking, a panGTPase inhibitor, CID-1067700, was used (38) Among targets of this compound are Rab proteins required for vesicular trafficking, for example, Rab7 (38, 54). Rab7 is a downstream target of the calmodulin pathway that regulates intracellular vesicular trafficking (55). We found that treating with IL-6 while blocking any one of these calmodulin pathway components produced a clear deficit in Fpn localization to the membrane and an increase in Fpn localized in the immediate intracellular space. This observation highlights the significance of calcium to the trafficking of Fpn, placing calcium as a mediator of IL-6 signaling and downstream transporter trafficking.

We also examined this IL-6 effect in hBMVEC, a widely used model of the blood brain barrier. Using ^55^Fe flux assays we determined that apical IL-6 treatment increased flux of ^55^Fe within 1 to 3 h, whereas at 6 h basal IL-6 decreased flux into the basal chamber. This behavior suggests that these barrier cells respond initially to inflammatory signals by accumulating iron, but a feedback loop is then induced to modulate transcellular iron flux. As mentioned, hepcidin binds to Fpn, inducing its recycling from the plasma membrane and delivery for ubiquitin-dependent degradation (9). Therefore, a hepcidin feedback loop may be initiated to bring cells back to iron homeostasis by preventing hyperaccumulation of iron. Indeed, we determined that hBMVEC hepcidin transcript increases in response to IL-6. We suggest that the IL-6/Ca-dependent trafficking of Fpn to the membrane precedes the induction of hepcidin and the subsequent removal of this Fpn from the membrane. This model is consistent with our finding that conditioned media from hBMVEC treated with IL-6 decreased the abundance of membrane Fpn in Fpn-GFP HEK cells. Lastly, we found that conducting flux experiments with basally treated hBVMEC in the presence of the hepcidin antagonist fursultiamine reversed the decrease in iron flux seen with IL-6 treatment alone. This suggests that the regulation of barrier Fpn by hepcidin is polarized in that it manifests at the basolateral but not apical membrane. We did not directly interrogate that possibility by direct observation, however. Overall, these experiments show that hBMVEC hepcidin can modulate the flux of iron across this barrier in response to IL-6 in combination with an IL-6–dependent plasma membrane localization of Fpn.

However, a key takeaway from this work is that Fpn localization can be regulated by mechanisms independent of hepcidin. In the general discussion of Fpn regulation, hepcidin and iron status are the dominant pathways discussed. Our work demonstrates that a rapid, transient response to IL-6 is involved in regulating Fpn membrane localization. When inducing an inflammatory state with IL-6 treatment, there is an increase in Fpn localization. This is unexpected as IL-6 induces hepcidin expression. Therefore, this further demonstrates that an initial response to inflammation regulates Fpn, prior to hepcidin initiation (10). It may also be possible that IL-6–dependent Fpn relocalization to the plasma membrane primes the transporter for hepcidin-mediated knockdown. This process occurs in response to iron loading, in which Fpn traffics to the plasma membrane where it is targeted for hepcidin regulation (30). We believe that the purpose of the IL-6 and calcium-mediated activation of Fpn is a protective response to inflammation that prepares the cell for removal of iron. This may be to make iron more available to macrophages and microglia and/or to remove excess iron that can be toxic to a cell, exacerbating the homeostasis of the cell prior to an inflammatory state.

In conclusion, we examined the role of IL-6 and downstream signaling in the regulation of Fpn plasma membrane localization and subsequent iron efflux. Both IL-6 and calcium treatment resulted in Fpn trafficking to the plasma membrane. Using calcium pathway inhibitors, we determined that ER calcium and calmodulin signaling are key to IL-6–induced Fpn trafficking. Inflammation is a feature of a multitude of chronic diseases, while iron metabolism has emerged as a key player as well. As most research into Fpn focuses on the Fpn–hepcidin axis, we provide novel insight with respect to Fpn relocalization initiated by IL-6. Overall, our data contributes to the connection between inflammatory processes and cellular iron metabolism, while shedding light on a new mechanism for Fpn regulation.

## Experimental procedures

### Cell culture

hBMVEC were a gift from Dr Supriya Mahajan (University at Buffalo); the generation and characteristics of this cell line have been described in detail and have been validated as an hBMVEC cell line (56, 57). hBMVEC were cultured as previously described (58), reaching about 90 to 95% confluency at the time of the experiment. HEK-293 cells were used as tool to better visualize movement of Fpn protein. Experiments in hBMVEC were tried, but it was difficult to visualize Fpn localization and the overall results were limited (Fig. S1). Therefore, Fpn-GFP HEK were the best and most robust option to answer these questions. Fpn-GFP HEK were a kind gift from Professor Elizabeta Nemeth (UCLA). Fpn-GFP HEK are a stably transfected cell line that are inducible by doxycycline using the Tet-on system as described in (32). Fpn-GFP HEK were cultured in calcium-free Dulbecco's modified Eagle's medium (DMEM) + 10% fetal bovine serum (Gibco), 2 mM L-glutamine, 1× Pen/Strep for 48 h prior to treatments.

For experiments examining calcium, cells were treated with 2 mM CaCl_2_ in calcium-free DMEM for 2 h. For experiments testing the effects of IL-6 (Peprotech), cells were treated with 100 ng/ml IL-6 in calcium-free DMEM. In experiments using XC (Tocris Biosciences), cells were treated with 3 μM XC in calcium-free DMEM. For experiments using calcium signaling inhibitors, cells were treated with 10 μM BAPTA (Invitrogen), 12.5 μM W-7 (MedChemExpress) 1 μM KN-93 (Cayman Chemicals), or 80 μM CID-1067700 (MedChemExpress) in calcium-free DMEM.

### Immunocytochemistry

Cells were seeded at 100K cells/well on sterile glass coverslips coated with 30 μg/ml bovine collagen (Advanced BioMatrix) in a 6-well plate. Cells were induced with 1 μg/ml doxycycline in calcium-free DMEM for 24 to 48 h post plating. After induction, cells were treated as described, then washed in PBS containing 0.5 mM MgCl_2_ (used throughout the procedure), and fixed for 10 min at room temperature in 3.7% paraformaldehyde and 4% sucrose in PBS. Cells were stained with Alexa Fluor 647–conjugated wheat germ agglutinin for 10 min in PBS as a cell surface marker. Cells were then washed twice in PBS, blocked, and permeabilized for 1 h at room temperature using 1% Bovine Serum Albumin (BSA), 0.3 M glycine, and 0.1% Tween-20 and then incubated with primary rabbit α-GFP antibody (Clontech, 632376, 1:2000) in 1% BSA overnight at 4 °C. The following day, coverslips were washed three times in PBS and incubated with secondary donkey anti-rabbit Alexa Fluor 488 antibody (Invitrogen, A21206, 1:1000) for 1 h in PBS with 1% BSA. Coverslips were washed three times in PBS, stained with 0.7 μg/ml Hoechst for 10 min as a nuclear stain, washed, and then mounted on glass microscope slides using Prolong Gold antifade mounting media (Invitrogen). Fpn-GFP/Alexa Fluor 488 (green), plasma membrane/wheat germ agglutinin Alexa Fluor 647 (red), nucleus/Hoechst 33342 (blue).

### Confocal microscopy

Images were obtained on the Leica TCS SP8 confocal microscope at 63× magnification with oil immersion. For GFP immunofluorescence, images were adjusted equally for brightness and quantified using ImageJ. Quantification was done using a line analysis method. Choosing a cell in an image, a line (0.68 μm) was drawn starting from outside the cell to a region inside the cell, placing the center point directly on the membrane. Histograms of the pixel intensity along the line were graphed, n = 12 regions of interest per image: four-line measurements across different regions of the membrane from three separate cells.

### Cell surface biotinylation

For biotinylation assays in hBMVEC and Fpn-GFP HEK, cells were treated with calcium or IL-6 for 1.5 h prior to biotinylation. Cells grown in 6-well plates were washed twice with PBS containing 0.5 mM MgCl_2_ (used for entire biotinylation protocol) and then treated with 0.5 mg/ml EZ-Link Sulfo-succinimidyl-2-(biotinamido)-ethyl-1,3-dithiopropionate-Biotin (Thermo Fisher Scientific) for 2 h at 4 °C. Then, cells were washed twice with PBS containing 0.1% BSA to quench unreacted biotin and washed twice with PBS. Cells were lysed by scraping in ice-cold radioimmunoprecipitation assay (RIPA) buffer (25 mM Tris, 150 mM NaCl, 1% NP-40, 1% Na-deoxycholate, 0.1% SDS, pH 7.4) supplemented with 4× Halt protease inhibitor cocktail (Thermo Fisher Scientific) and incubated on ice for 15 min. The cell suspension was centrifuged at 10,000*g* for 10 min at 4 °C, and the supernatant (input fraction) was collected. Protein content was quantified and equal amounts (300–500 μg) were loaded onto a NeutrAvidin agarose column (Thermo Fisher Scientific) overnight at 4 °C. Columns were washed with RIPA buffer containing 4× protease inhibitors and eluted at 50 °C in 6× SDS-loading buffer, 150 mM DTT (bound fraction). Equal amounts of input protein (20 μg) and the bound fraction elution were loaded into each lane for SDS-PAGE, followed by Western blotting. Western blots were probed for α-GFP (Clontech, 632376, 1:2000) α-β-actin (Cell Signaling, 8457, 1:1000) and α-Fpn (Novus Biologicals, NBP1-21502, 1:1000).

### ^55^Fe efflux assays

For iron efflux assays, cells were loaded with 1 μM ^55^Fe (Oak Ridge National Laboratory) for 24 h prior to treatment. Pretreatments are added in the ^55^Fe-containing media (calcium-free DMEM + 10% FBS) during the iron loading step just prior to efflux and then efflux was initiated by removal of the ^55^Fe-containing media and adding media containing efflux treatments. At the start of efflux (t = 0) and after efflux (t = 2 h), cells were lysed with RIPA buffer and samples were counted for ^55^Fe content by liquid scintillation counting. ^55^Fe counts were normalized to protein content quantified by bicinchoninic acid assay (Thermo Fisher Scientific). Iron accumulation was expressed as pmol ^55^Fe per mg protein remaining in cell lysates, and efflux was calculated as the percent loss of ^55^Fe compared to the amount of ^55^Fe in cell lysates at t = 0 for each treatment group.

### ^55^Fe flux assays

For iron flux assays, hBMVEC were plated in the apical chamber of transwell inserts in Roswell Park Memorial Institute + serum media. For 2 days following, *trans*-endothelial electrical resistance measurements were taken to ensure proper barrier formation. The day after plating, media in the basal chamber was changed to Roswell Park Memorial Institute – serum to polarize the cells. On experimental day, IL-6 was added either in the apical chamber or the basal chamber for 2 h. ^55^Fe (1 μM) was added to the apical or basal chamber of the transwell and cells were incubated for 6 h. Media from the apical or basal chamber was collected at 1, 3, and 6 h. After 6 h, cells were lysed. Lysates and media samples were counted for ^55^Fe using a liquid scintillation counter. Lysates were used to confirm equivalent protein content by bicinchoninic acid assay. Iron flux was expressed as pmol ^55^Fe in the apical or basal media at each timepoint, accounting for the removal of media and ^55^Fe at all previous timepoints.

### Statistical analysis

Statistical analyses were performed using Prism 8.0 or 9.0 (GraphPad Software, https://www.graphpad.com/features). Data are presented as scatter plots with mean ± SEM unless otherwise noted. Unpaired *t* tests were used when comparisons were made between two conditions (one variable) from the same time point. Comparisons of multiple samples were made using one-way ANOVA statistical analyses, specifics noted below. In the text, the number of biologic and technical replicates were indicated and used to derive the stated quantity. To analyze Fpn-GFP intensity from the confocal microscopy images and the ImageJ line method, values were first limited between 0.3 and 0.36 μm, ±0.03 μm from the plasma membrane peak (0.33 μm). Another set of measurements were taken limiting values between 0.3 and 0.68 μm, to represent further inside the membrane. Then, background corrections were made. Three lines (0.68 μm) from each image were made in regions of no fluorescence, averaged, and subtracted from the appropriate condition using GraphPad Prism. The data was fit using nonlinear regression analysis. Then, AUC was calculated, and statistical significance was determined using one-way ANOVA with the Brown–Forsythe test for unequal standard deviations and Games-Howell tests for n >50. The AUC measurement accounts for every data point along the line and then compiles that data from all 12 lines into the reported AUC. GraphPad Prism reports that as one mean ± SEM with the n value represented by all of the data points. We report this as a bar graph with the mean and SEM.

For iron efflux assays, statistical significance was determined using unpaired *t* test for comparing two treatment groups: ∗∗*p* < 0.01 and ∗∗∗∗*p* < 0.0001. For experiments with three treatment groups, statistical significance was determined using one-way ANOVA with Tukey’s multiple comparison test; *ns*, not statistically significant; ∗*p* < 0.05; and ∗∗*p* < 0.01.

## Data availability

All relevant data are contained within this article.

## Supporting information

This article contains supporting information.

## Conflict of interest

The authors declare that they have no conflicts of interest with the contents of this article.
