# Supplementary material for: Calcium and IL-6 regulate the anterograde trafficking and plasma membrane residence of the iron exporter ferroportin to modulate iron efflux
Source: J Biol Chem. 2024 May 6;300(6):107348. doi: 10.1016/j.jbc.2024.107348 (PMC11154712; doi:10.1016/j.jbc.2024.107348)
Supplement: Figure S1, Tables S1 and S2 [file mmc1.pdf]

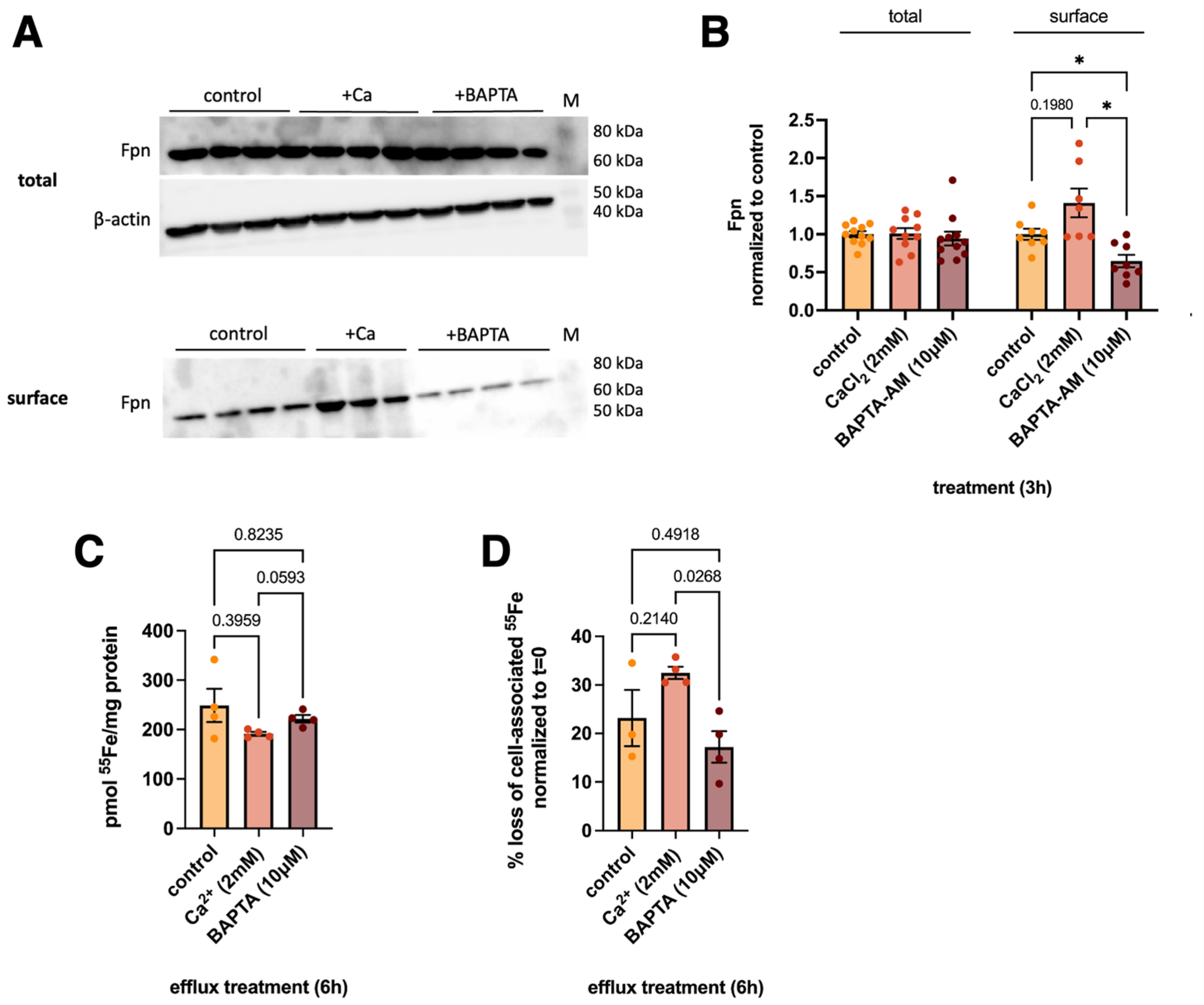

Figure S1. Calcium increases FPN surface protein residence and Fe efflux in human brain microvascular endothelial cells. Cells were treated with 2 mM $CaCl_2$ or 100 ng/ml IL-6 for 1.5 h prior to biotinylation. After treatment, cell surface proteins were labeled with sulfo-NHS-SS-biotin, cell were lysed, and protein content was quantified. Equal amounts of protein were loaded on Neutravidin columns and incubated overnight, then bound protein was eluted in SDS loading buffer containing 150 mM DTT. ***A,*** Western blots for Fpn or β-actin in total and surface fractions. M, Magic Mark. ***B,*** Band intensities for FPN were normalized to those for β-actin, and quantification of the band intensities relative to control are shown for both total and surface fractions. This experiment includes 3 trials with 3-6 replicates per condition. Statistical significance was tested by one-way ANOVA for total or bound fractions independently. P-values are noted. Cells were loaded with 1 uM ^{55}Fe for 24 h prior to efflux. To initiate efflux, ^{55}Fe-containing media was removed and cells were incubated in media containing 2mM $CaCl_2$ or 10 µM BAPTA for 6 h. After 6 h, cells are lysed and samples counted for ^{55}Fe content by liquid scintillation counting. ***C,*** ^{55}Fe content normalized to protein content is reported. ***D,*** Percent loss of ^{55}Fe from Fpn-GFP HEK, normalized to protein content and represented as percent of t = 0 for that treatment group. N = 4 biological replicates/condition. Statistical significance was tested by one-way ANOVA. All p-values are reported.

Table S1

**Games-Howell's Multiple Comparison Test**	**p-value**
control vs. +IL-6 3hrs	<0.0001
control vs. +BAPTA +IL-6	<0.0001
control vs. +W7 +IL-6	<0.0001
control vs. +KN-93 +IL-6	<0.0001
control vs. +CID +IL-6	<0.0001
+IL-6 3hrs vs. +BAPTA +IL-6	<0.0001
+IL-6 3hrs vs. +W7 +IL-6	>0.9999
+IL-6 3hrs vs. +KN-93 +IL-6	0.2805
+IL-6 3hrs vs. +CID +IL-6	<0.0001
+BAPTA +IL-6 vs. +W7 +IL-6	0.0003
+BAPTA +IL-6 vs. +KN-93 +IL-6	<0.0001
+BAPTA +IL-6 vs. +CID +IL-6	0.1556
+W7 +IL-6 vs. +KN-93 +IL-6	0.5211
+W7 +IL-6 vs. +CID +IL-6	<0.0001
+KN-93 +IL-6 vs. +CID +IL-6	<0.0001

Table S1. p-values for Games-Howell's Multiple Comparisons Test of Figure 7. The area under the curve (AUC) was analyzed for values from 0.3 to 0.36 μm and quantified using one-way ANOVA with a Games-Howell post-hoc test. The experiment was completed once with n=4 lines per cell (technical replicates) and n=3 cells (biological replicates) per condition. p-values of each comparison made are shown for complete clarity.

Table S2

**Games Howell's Multiple Comparison Test**	**p-value**
control vs. +IL-6 3hrs	<0.0001
control vs. +BAPTA +IL-6	<0.0001
control vs. +W7 +IL-6	<0.0001
control vs. +KN-93 +IL-6	<0.0001
control vs. +CID +IL-6	<0.0001
+IL-6 3hrs vs. +BAPTA +IL-6	<0.0001
+IL-6 3hrs vs. +W7 +IL-6	<0.0001
+IL-6 3hrs vs. +KN-93 +IL-6	<0.0001
+IL-6 3hrs vs. +CID +IL-6	<0.0001
+BAPTA +IL-6 vs. +W7 +IL-6	<0.0001
+BAPTA +IL-6 vs. +KN-93 +IL-6	<0.0001
+BAPTA +IL-6 vs. +CID +IL-6	<0.0001
+W7 +IL-6 vs. +KN-93 +IL-6	<0.0001
+W7 +IL-6 vs. +CID +IL-6	<0.0001
+KN-93 +IL-6 vs. +CID +IL-6	<0.0001

Table S2. p-values for Games-Howell's Multiple Comparisons Test of Figure 7. The area under the curve (AUC) was analyzed for values from 0.3 to 0.68 μm and quantified using one-way ANOVA with a Games-Howell post-hoc test. The experiment was completed once with n=4 lines per cell (technical replicates) and n=3 cells (biological replicates) per condition. p-values of each comparison made are shown for complete clarity.
